# Transitioning from conventional radiotherapy to intensity-modulated radiotherapy for localized prostate cancer: changing focus from rectal bleeding to detailed quality of life analysis

**DOI:** 10.1093/jrr/rru061

**Published:** 2014-09-08

**Authors:** Hideya Yamazaki, Satoaki Nakamura, Takuya Nishimura, Ken Yoshida, Yasuo Yoshioka, Masahiko Koizumi, Kazuhiko Ogawa

**Affiliations:** 1 Department of Radiology, Graduate School of Medical Science, Kyoto Prefectural University of Medicine, 465 Kajiicho Kawaramachi Hirokoji, Kamigyo-ku, Kyoto 602-8566, Japan; 2 Department of Radiology, Osaka Medical College, 2-7 Daigaku-machi, Takatsuki-City, Osaka, 569-8686, Japan; 3 Department of Radiation Oncology, Osaka University Graduate School of Medicine, Yamadaoka 2-2, Suita, 565-0871 Osaka, Japan

**Keywords:** prostate cancer, radiotherapy, rectal bleeding, incontinence, genitourinary symptom, erectile dysfunction

## Abstract

With the advent of modern radiation techniques, we have been able to deliver a higher prescribed radiotherapy dose for localized prostate cancer without severe adverse reactions. We reviewed and analyzed the change of toxicity profiles of external beam radiation therapy (EBRT) from the literature. Late rectal bleeding is the main adverse effect, and an incidence of >20% of Grade ≥2 adverse events was reported for 2D conventional radiotherapy of up to 70 Gy. 3D conformal radiation therapy (3D-CRT) was found to reduce the incidence to ∼10%. Furthermore, intensity-modulated radiation therapy (IMRT) reduced it further to a few percentage points. However, simultaneously, urological toxicities were enhanced by dose escalation using highly precise external radiotherapy. We should pay more attention to detailed quality of life (QOL) analysis, not only with respect to rectal bleeding but also other specific symptoms (such as urinary incontinence and impotence), for two reasons: (i) because of the increasing number of patients aged >80 years, and (ii) because of improved survival with elevated doses of radiotherapy and/or hormonal therapy; age is an important prognostic factor not only for prostate-specific antigen (PSA) control but also for adverse reactions. Those factors shift the main focus of treatment purpose from survival and avoidance of PSA failure to maintaining good QOL, particularly in older patients. In conclusion, the focus of toxicity analysis after radiotherapy for prostate cancer patients is changing from rectal bleeding to total elaborate quality of life assessment.

## INTRODUCTION

Prostate cancer is one of the most prevalent solid tumors diagnosed in men in the USA and developed countries. Recent research in numerous randomized controlled trials demonstrated that increasing the prescribed dose in the treatment of localized prostate cancer improves biochemical control in several risk categories: low-, intermediate- and high-risk prostate cancer patients, at least for certain subgroups of patients, as summarized in two recent meta-analyses [1, 2] (Table 1). Consequently, the National Comprehensive Cancer Network Clinical Practice Guidelines in Oncology (2013) state that doses of 75.6–79.2 Gy in conventional fractions delivered to the prostate are appropriate for patients with low-risk cancers. For patients with intermediate- or high-risk disease, a dose of up to 81.0 Gy provides improved prostate-specific antigen (PSA)-assessed disease control [3].

**Table 1.** Conventional radiation therapy and 3D conformal radiation (3D-CRT) therapy

Author (Institute)	Year (Pt No.)	Study	Follow-up (median)	Radiotherapy	PSA control rate* (L/I/H)	Adverse toxicity criteria	Adverse reaction Late G2 or more if otherwise cited	
**Conventional 2D vs 3D-CRT**								
Dearnaley [5] (UK)	1999 (*n* = 225)	RCT: 2D vs 3D-CRT *n* = 111 vs 114	3.6 years	64 Gy	3.6 years 78% vs 83%	RTOG	GI 15% vs 5% *P* = 0.01 GU 23% vs 20%	3D-CRT reduced GI toxicity
Koper [6] (Netherland)	2004 (*n* = 248)	RCT: 2D vs 3D-CRT *n* = 125 vs 123	2 years (minimum)	66 Gy	NA	modified score	late rectum 10% vs 7%, anus 2% vs 2%, bladder 11% vs 9%	3D-CRT ≒ 2D at 66 Gy pre-existing/acute symptoms related to late reaction
Yoshioka [7] (Osaka Univ.)	2013 (*n* = 362)	2D vs 3D-CRT *n* = 127 vs 235	4.5 years	70 Gy	NA	CTCAE v 4.0	GI 23% vs 7% *P* < 0.001	3D-CRT reduced field widths and GI toxicity
**3D-CRT**								
Kuban [8] (MDAC)	2008 (*n* = 300)	RCT *n* = 149 vs 151	8.7 years	70 Gy vs 78 Gy	8 years 50% vs 73% *P* = 0.004 (63%/76%/26%) vs (88%/86%/63%)	RTOG/LENT	GI 13% vs 26% *P* = 0.013 GU 8% vs 13%	higher dose improved PSA control and elevated GI toxicity
Zietman [9] (MGH)	2005 (*n* = 392)	RCT *n* = 197 vs 195	5.5 years	70.2 GyE vs 79.2 GyE 3D-CRT 50.4 Gy + Proton Boost 28.8 GyE vs 19.8 GyE	61.4% vs 80.4% *P* < 0.001	RTOG	GI 9% vs 18% *P* = 0.005 GU 20% vs 21%	higher dose improved PSA control and elevated toxicity
Peeters [10] (Netherland)	2006 (*n* = 664)	RCT: Dutch trial *n* = 331 vs 333	51 months	68 Gy vs 78 Gy	54% vs 64% *P* = 0.02	RTOG/EORTC modified	GI 27% vs 32% GU 39% vs 41%	higher dose improved PSA control higher dose elevated GI toxicity (25% vs 35%) at 7 years [11]
Dearnaley [12] (UK)	2007 (*n* = 843)	RCT: MRC RT01 *n* = 421 vs 422	5 years	64 Gy vs 74 Gy	60% vs 71% *P* = 0.000 7	RTOG	GI 24% vs 33% *P* = 0.005 GU 8% vs 11%	higher dose improved PSA control and elevated GI toxicity
Skwarchuk [13] (MSK)	2000 (*n* = 743)	Dose escalation *n* = 96 vs 266 vs 320 vs 61	5 years	64.8 Gy vs 70.2 Gy vs 75.6 Gy vs 81 Gy	NA	RTOG/EORTC modified LENT/SOMA	GI 3.4% vs 7.8% vs 15.9% vs 16.5%	higher dose elevated GI toxicity
Pollack [14] (MDAC)	2002 (*n* = 301)	RCT *n* = 150 vs 151	6 years	70 Gy vs 78 Gy	6 years 64% vs 70% *P* = 0.03	RTOG	rectum 12% vs 26% *P* = 0.001 bladder both 10%	higher dose improved PSA control and elevated GI toxicity
Michalsky [15] (RTOG 9406)	2010 (*n* = 1084)	Dose escalation *n* = 112 vs 300 vs 167 vs 256 vs 220	6.1–12.1 years	68.4 Gy vs 73.8 Gy vs 79.2 Gy vs 74 Gy vs 78 Gy	NA	RTOG	GI: 9% vs 7% vs 11% vs 10% vs 25% (#Group 1) *P* = 0.0001 GI 13% vs 9% vs 14% vs 16% vs 26% (#Group 2) *P* = 0.0063 GU 16–29%	Higher dose elevated GI toxicity
Beckendorf [16] (France)	2011 (*n* = 306)	RCT: GETUG *n* = 153 each	61 months	70 Gy vs 80 Gy	61% vs 72% *P* = 0.03	RTOG modified	GI 14% vs 19.5% GU 10% vs 17.5% *P* = 0.046	higher dose improved PSA control with elevated urinary toxicity

MDAC = MD Anderson Cancer Center, MGH = Massachusetts General Hospital, MSK = Memorial Sloan-Kettering Cancer Center, 2D = conventional radiotherapy, NA = not available, RCT = radomized controlled trial, CTCAE = Common Terminology Criteria for Adverse Events, RTOG = Radiation Therapy Oncology Group, EORTC = European Organization for Research and Treatment of Cancer late morbidity, LENT/SOMA = Late Effect Normal Tissues/Subjective, Objective, Management, and Analytic, L/I/H = low risk/intermediate risk/high risk groups, GI = gastrointestinal, GU = genitourinary *5 years unless otherwise stated, #Group 1 treated for prostate only and Group 2 for seminal vesicle and prostate.

On the other hand, survival was at least as good as that expected for an age-matched group of patients from the general population [4]. The fact that elderly patients will die should be considered, if not from their prostate cancer, then from one of the many competing causes of death. Therefore, it is important to determine what could most likely cause their demise. In high-risk patients who are relatively younger (<70 years old at diagnosis), dose escalation leads to a much higher likelihood of dying of a cause other than cancer. Perhaps equally notable, patients who are aged >70 years during treatment never die of prostate cancer when the dose is escalated to 78 Gy or with hormonal treatment [4]. These accomplishments in outcome must be weighed against the complication rate. Fortunately, technology and parameters for dose restriction to normal tissues have provided measures to ensure that the therapeutic index remains high. In this document, we attempted to review the change in toxicity profiles from 2D radiation to the era of image-guided radiotherapy in the face of a dramatic increase in the number of older patients. We analyzed the changing trends in adverse effects of external beam radiotherapy (EBRT). Although there are many good outcomes of brachytherapy (BT) for localized prostate cancer, to keep the analysis simple we did not include BT. The PubMed database was searched for relevant articles published after 1990. We included only studies published in English assessing adverse effects in patients following curative EBRT that had large sample sizes (more than 100 patients) and/or important findings.

## LITERATURE REVIEW

### From conventional (2D) radiotherapy to 3D conformal radiotherapy

Standard 2D planning techniques used until the 1990s with limited total doses of up to 70 Gy were expected to cause toxicity. In the 1990s, 3D planning techniques were developed, and 3D conformal radiation therapy (3D-CRT) was combined with computer software to integrate CT images of the patient's internal anatomy. These approaches allowed physicians to work with a high-dose irradiated volume. The role of dose escalation has been estimated in several randomized controlled trials, and the results indicate that a higher dose improves PSA control with elevated toxicity, mainly in the form of rectal bleeding [1, 2, 5–16] (Table 1). Most of the evidence of late radiation toxicity comes from those 3D-CRT dose escalation studies.

Dearnaley *et al*. conducted a randomized controlled trial to compare the toxicity of 2D with 3D-CRT with a standard dose of 64 Gy in daily 2-Gy fractions and concluded that conformal techniques significantly lower the risk of late radiation-induced proctitis after radiotherapy for prostate cancer [5]. In the 225 men treated, significantly fewer men developed radiation-induced proctitis and bleeding in the conformal group than in the conventional group (37% vs 56% ≥ Radiation Therapy Oncology Group (RTOG) Grade 1, *P* = 0.004; 5% vs 15% RTOG ≥ Grade 2, *P* = 0.01). There were no differences between the groups with respect to bladder function after treatment (53% vs 59% ≥ Grade 1, *P* = 0.34; 20% vs 23% ≥ Grade 2, *P* = 0.61). After a median follow-up period of 3.6 years, there was no significant difference between the groups in local tumor control.

Koper *et al*. reported that conformal radiotherapy at a dose level of 66 Gy does not significantly decrease the incidence of gastrointestinal (GI) rectal (10% vs 7%), anal and genitourinary (GU) bladder toxicity compared with conventional radiotherapy in a Phase 3 trial [6]. There is a significant relationship between acute and late toxicity and the anal volume exposed to 90% of the tumor dose. GI and GU symptoms at the start have a major impact on late toxicity.

Yoshioka *et al*. compared late toxicity for 2D- with 3D-CRT using uniform radiotherapy of 70 Gy in 35 fractions, employing the classical four-field technique with gantry angles of 0°, 90°, 180° and 270° in 362 patients at five institutions with a median follow-up of 4.5 years (range, 1.0–11.6) [7]. The 5-year overall and cause-specific survival rates were 93% and 96%, respectively. The mean ± SD of portal field size in the right–left, superior–inferior and anterior–posterior directions was 10.8 ± 1.1, 10.2 ± 1.0 and 8.8 ± 0.9 cm for a 2D simulation and 8.4 ± 1.2, 8.2 ± 1.0 and 7.7 ± 1.0 cm for a 3D simulation (*P* < 0.001), respectively. No Grade 4 or 5 late toxicity was observed. The actuarial 5-year Grade 2–3 GU and GI late toxicity rates were 6% and 14% respectively, whereas the corresponding late rectal bleeding rate was 23% for a 2D simulation and 7% for a 3D simulation (*P* < 0.001). The use of a CT simulation and the resultant reduction in portal field size were significantly associated with reduced late GI toxicity, and particularly with less rectal bleeding.

Consequently, several dose escalation studies have been conducted (Table 1) [8–16]. Viani *et al*. performed a meta-analysis of seven randomized controlled trials with a total patient population of 2812 [1]. Pooled results from these studies showed a significant reduction in the incidence of biochemical failure in patients with prostate cancer treated with high-dose radiotherapy (*P* < 0.0001). On the other hand, there was no difference in the mortality rate (*P* = 0.38) or in specific prostate cancer mortality rates (*P* = 0.45) between the groups receiving high-dose radiotherapy and conventional-dose radiotherapy. Nevertheless, there were more cases of late Grade >2 GI toxicity after high-dose radiotherapy than after conventional dose radiotherapy. In the subgroup analysis, patients classified as being at a low (*P* = 0.007), intermediate (*P* < 0.0001), and high risk (*P* < 0.0001) of biochemical failure all showed a benefit from high-dose radiation therapy.

### From 3D-CRT to intensity-modulated radiotherapy

A further advancement in radiotherapy techniques that facilitates precise dose delivery is intensity-modulated radiation therapy (IMRT). This technique allows dose escalation while minimizing damage to the normal tissue (Table 2) [17–25].

**Table 2.** 3D conformal radiation therapy (3D-CRT) and intensity-modified radiation therapy (IMRT)

Author (Institute)	Year (Pt No.)	Study	Follow-up period (median)	Radiotherapy	PSA control rate* (L/I/H)	Adverse toxicity criteria	Adverse reaction Late G2 or more if otherwise cited	
**3D-CRT vs IMRT**								
Zelefsky [17] (MSK)	2008 (*n* = 1571)	3D-CRT vs IMRT n = 830 vs 741	10 years	3D-CRT vs IMRT 66-75.6 Gy vs 81 Gy	NA	CTCAE ver. 3.0	GI 13% vs 5% *P* ≤ 0.001 GU 20% vs 12% *P* = 0.01	IMRT reduces GI but increases GU toxicity Acute related to late toxicity
Vora [18] (Mayo)	2007 (*n* = 416)	3D-CRT vs IMRT *n* = 271 vs 145	5 years	3D-CRT vs IMRT 68.4 (66–71) Gy vs 75.6 (70.2–77.4) Gy	74.4% vs 84.6% *P* = 0.032 6	CTCAE ver. 4.0	GI 16% vs 24% GU 29% vs 22%	high dose IMRT improved PSA control in intermediate and high risk groups
Sharma [19] (Fox Chase)	2011 (*n* = 293)	3D-CRT + ADT vs IMRT + ADT *n* = 170 vs 123	86 months vs 40 months		NA	Fox chase modified LENT	GI 20% vs 8% *P* = 0.01 GU 6.5% vs 4.8%	IMRT reduced GI toxicity
Bekekman [20] (UPEN)	2011 (*n* = 12 598)	3D-CRT vs IMRT *n* = 6753 vs 5845	24 months SEER–Medicare database	NA aged 65 years or older	NA	Medicare patient claim composite bowel complication	bowel 22.5% vs 18.8%; HR 0.86 proctitis/hemorrhage; HR 0.78	IMRT slightly reduced GI toxicity
Sheets [21] (North Carolina)	2012 (*n* = 12 976)	3D-CRT vs IMRT (vs proton) *n* = 6753 vs 5845 vs 1368	44 months vs 64 months and 46 months vs 50 months SEER–Medicare database	NA (propensity score–adjusted analyses)	NA	Medicare patient claim	GI 14.7 vs 13.4 per 100 person-years Hip fracture 1.0 vs 0.8, ED 5.3 vs 5.9	IMRT less GI toxicity and hip fractures, more ED than 3D-CRT (IMRT less GI toxicity than proton 12.2 vs 17.8)
Michalsky [22] (RTOG 0126)	2013 (*n* = 748)	RCT: 3D-CRT vs IMRT *n* = 491 vs 257	4.6 years vs 3.5 years	79.2 Gy	NA	CTC ver. 2.0 RTOG/EORTC	GI 22% vs 15.1% *P* = 0.039 GU NA	IMRT reduced GI toxicity but not significant in multivariate analysis
**IMRT**								
Alicikus [23] (MSK)	2011 (*n* = 170)	Long-term follow-up	99 months	81 Gy	10 years (81%/78%/62%)	CTCAE ver. 3.0	GI 3% GU 16%	99 months long-term results
Spratt [24] (MSK)	2013 (*n* = 1 002)	High-dose IMRT	5.5 years	86.4 Gy	7 years (99%/86%/68%)	CTCAE ver. 4.0	GI 4.4% GU 21.1%	86.4 Gy feasible
Pederson [25] (Chicago)	2012 (*n* = 296)	Dose constraint assessment	41 months	76 Gy	NA	CTCAE ver. 3.0	GI 5% GU 9%	Whole-pelvic IMRT related to GU toxicity, age to GI GI 0% if V70 ≤ 10%, V65 ≤ 20%, and V40 ≤ 40%

MSK = Memorial Sloan-Kettering Cancer Center, UPEN = University of Pennsylvania, EORTC = European Organization for Research and Treatment of Cancer, RCT = radomized controlled trial, NA = not available, CTC = Common Toxicity Criteria, CTCAE = Common Terminology Criteria for Adverse Events, RTOG; Radiation Therapy Oncology Group, GI gastrointestinal, GU; genitourinary, ED = erectile dysfunction, HR = hazard risk, SEER = Surveillance, Epidemiology and End Results, LENT/SOMA = Late Effect Normal Tissues/Subjective, Objective, Management, and Analytic, (L/I/H) = (low risk/intermediate risk/high risk groups), *5 years unless otherwise stated.

Zelefsky *et al*. compared outcomes between 830 3D-CRT and 741 IMRT treatments and concluded that serious late toxicity is unusual, despite the delivery of high radiation doses from 66–81 Gy with a median follow-up of 10 years [17]. Higher doses were associated with increased GI and GU Grade 2 toxicity, but the risk of proctitis was significantly reduced with IMRT. Acute symptoms were a precursor of late toxicity in these patients. After 10 years, the actuarial likelihood of the development of ≥ Grade 2 GI toxicity was 9%. The use of IMRT significantly reduced the risk of GI toxicity compared with patients treated with conventional 3D-CRT (from 13% to 5%; *P* < 0.001). Among patients who experienced acute GI symptoms, the 10-year incidence of late toxicity was 42%, compared with 9% in those who did not experience acute symptoms (*P* < 0.0001). The 10-year incidence of late Grade ≥ 2 GU toxicity was 15%. Patients treated with 81 Gy IMRT had a 20% incidence of GU symptoms 10 years later, compared with 12% in patients treated with lower doses (*P* = 0.01). From the same institute, Spratt *et al*. reported results from a large cohort of 1002 patients treated with high-dose radiation of 86.4 Gy with a median follow-up period of 5.5 years (range, 1–14 years) [18]. A total of 587 patients (59%) were treated with neoadjuvant and concurrent androgen deprivation therapy (ADT). For low-, intermediate- and high-risk groups, 7-year biochemical relapse-free survival outcomes were 98.8%, 85.6% and 67.9%, respectively (*P* < 0.001). The incidence of actuarial 7-year Grade ≥2 late GI and GU toxicity was 4.4% and 21.1%, respectively. Late Grade 3 GI and GU toxicity was experienced by seven patients (0.7%) and 22 patients (2.2%), respectively.

Vora *et al*. reported an improved PSA control rate as a result of high-dose IMRT compared with conventional-dose 3D-CRT without elevated toxicity. A total of 416 patients with a minimum follow-up of 3 years (median 5 years) were included [18]. Of these, 271 patients received 3D-CRT with a median dose of 68.4 Gy (range, 66–71 Gy). Next, 145 patients received IMRT with a median dose of 75.6 Gy (range, 70.2–77.4 Gy). The 5-year biochemical control rate was 74.4% and 84.6% with 3D-RT and IMRT, respectively (*P* = 0.0326). The high-dose IMRT group experienced greater acute GU toxicity (*P* = 0.094) than the 3D-CRT group, but the difference was not statistically significant. There were no differences in acute GI (*P* = 0.83), chronic GU (*P* = 0.33), and chronic GI (*P* = 0.24) toxicity between the two groups.

Sharma *et al*. reported that IMRT + ADT reduced GI toxicity compared with 3D-CRT + ADT [19]. ADT has been shown to increase late Grade ≥2 rectal toxicity when used concurrently with 3D-CRT. A total of 293 men underwent 3D-CRT (*n* = 170) or IMRT (*n* = 123) with concurrent ADT (<6 months, *n* = 123; ≥6 months, *n* = 170). The median radiation dose was 76 Gy for 3D-CRT and 76 Gy for IMRT. Toxicity was assessed using a patient symptom questionnaire using a Fox Chase Modified Late Effect Normal Tissues (LENT) scale. The mean follow-up period was 86 months for the 3D-CRT group and 40 months for the IMRT group. The acute GI toxicity (odds ratio [OR], 4; 95% confidence interval [CI], 1.6–11.7; *P* = 0.005) was significantly greater with 3D-CRT than with IMRT and was independent of the ADT duration (i.e. <6 vs ≥6 months). The time to development of late GI toxicity was significantly longer in the IMRT group. The 5-year estimated incidence of Grade ≥2 GI toxicity was 20% for 3D-CRT and 8% for IMRT (*P* = 0.01). In multivariate analysis, Grade ≥2 late GI toxicity [hazard ratio (HR), 2.1; 95% CI, 1.1–4.3; *P* = 0.04) was more prevalent among the 3D-CRT-treated patients.

Bekelman *et al*. conducted an observational cohort study using data on registry and administrative claims from the Surveillance, Epidemiology and End Results (SEER)–Medicare database for patients aged ≥65 years diagnosed with non-metastatic prostate cancer in the USA who received IMRT (*n* = 5845) or CRT (*n* = 6753) [20]. IMRT was associated with a reduction in composite bowel complications (24-month cumulative incidence 18.8% vs 22.5%; HR, 0.86; 95% CI, 0.79–0.93) and proctitis/hemorrhage (HR, 0.78; 95% CI, 0.64–0.95). IMRT use was not associated with higher rates of composite urinary complications [HR, 0.93; 95% CI, 0.83–1.04) or cystitis/hematuria (HR, 0.94; 95% CI, 0.83–1.07). The incidence of erectile dysfunction (ED) involving invasive procedures was low and did not differ significantly between the groups, although IMRT was associated with an increase in new diagnoses of ED (HR, 1.27; 95% CI, 1.14–1.42). Those authors concluded that IMRT is associated with a small reduction in composite bowel complications and proctitis/hemorrhage compared with CRT in elderly men with non-metastatic prostate cancer.

Sheets *et al*. reported that the use of IMRT vs CRT increased from 0.15% in 2000 to 95.9% in 2008 [21]. In propensity score-adjusted analysis (*P* = 12 976), men who received IMRT vs CRT were less likely to receive a diagnosis of GI morbidity (absolute risk, 13.4 vs 14.7 per 100 person-years; relative risk [RR], 0.91; 95% CI, 0.86–0.96) or a hip fracture (absolute risk, 0.8 vs 1.0; RR, 0.78; 95% CI, 0.65–0.93), but more likely to receive a diagnosis of ED (absolute risk, 5.9 vs 5.3; RR, 1.12; 95% CI, 1.03–1.20).

Recently, Michalsky *et al*. reported preliminary toxicity analysis of 3D-CRT versus IMRT on the high-dose arm of the RTOG 0126 prostate cancer trial [22]. Of 763 patients randomized to the 79.2 Gy arm, 748 were eligible and evaluable: 491 and 257 were treated with 3D-CRT and IMRT, respectively. For both bladder and rectum, the volumes receiving 65, 70 and 75 Gy were significantly lower with IMRT (for all *P* < 0.0001). For Grade ≥2 acute GI/GU toxicity, both univariate and multivariate analysis showed a statistically significant decrease in Grade ≥2 acute collective GI/GU toxicity for IMRT. There were no significant differences between 3D-CRT and IMRT in acute or late Grade ≥2 or Grade ≥3 GU toxicity. In multivariate analysis, IMRT showed a 26% reduction in Grade ≥2 late GI toxicity (*P* = 0.099). Acute Grade ≥2 toxicity was associated with late Grade ≥3 toxicity (*P* = 0.005). RT modality was not significant, whereas white race (*P* = .001) and rectal V70 ≥15% were associated with G2+ rectal toxicity (*P* = 0.034). Thus, IMRT is associated with a significant reduction in acute Grade ≥2 GI/GU toxicity. There is a trend for a clinically meaningful reduction in late Grade ≥2 GI toxicity with IMRT. The occurrence of acute GI toxicity and large (>15%) volumes of rectum >70 Gy are associated with late rectal toxicity.

Ariskus *et al*. assessed long-term tumor control and toxicity outcomes after high-dose IMRT in 170 patients who received 81 Gy with a median follow-up period of 99 months [23]. The 10-year PSA control rates were 81% for the low-risk group, 78% for the intermediate-risk group, and 62% for the high-risk group. The 10-year cause-specific mortality rates were 0%, 3% and 14%, respectively. The 10-year likelihood of developing Grade 2 and 3 late GU toxicity was 11% and 5%, respectively; and the 10-year likelihood of developing Grade 2 and 3 late GI toxicity was 2% and 1%, respectively.

To our knowledge, only one manuscript dealt with the constraints of IMRT, but the data were not significant in multivariate analysis. Pederson *et al*. reported that a 4-year absence of maximal Grade ≥2 late toxicity is observed in 81% and 91% of patients in terms of GU and GI symptoms respectively, with a median follow-up period of 41 months after 76 Gy of IMRT [25]. In multivariate analysis, whole-pelvis IMRT was associated with Grade ≥2 GU toxicity, and age was associated with Grade ≥ 2 GI toxicity. The absence of Grade ≥ 2 GI toxicity after 4 years was observed in 100% of men with rectal V70 ≤ 10%, V65 ≤ 20% and V40 ≤ 40%; 92% of men with rectal V70 ≤ 20%, V65 ≤ 40% and V40 ≤ 80%; and 85% of men exceeding these criteria (*P* = 0.13). These criteria were more strongly associated with GI toxicity in men aged ≥70 years (*P* = 0.07). At present, no confirmed constraints exist in IMRT, and further studies are required.

### From IMRT to image-guided radiation therapy

Image-guided radiation therapy (IGRT) is the process of frequent 2D and 3D imaging, in the course of a radiation treatment, intended to direct radiation therapy using imaging coordinates of the actual radiation treatment plan. This approach allows physicians to deliver accurate radiation therapy with a reduction in the set-up margin (Table 3) [26–31].

**Table 3.** Intensity modulated radiation therapy (IMRT) and image guided radiation therapy (IGRT)

Author (Institute)	Year (Pt No.)	Study IGRT methods	Follow-up period (median)	Radiotherapy	PSA control rate* (L/I/H)	Adverse toxicity criteria	Adverse reaction Late Grade 2 or more if otherwise cited	
**IMRT vs IG-IMRT**								
Zelefsky [26] (MSK)	2012 (*n* = 376)	IMRT vs IG-IMRT CBCT, Fiducial *n* = 190 vs 186	2.8 years	86.4 Gy	High-risk group (*n* = 67 vs 35) 3 years 77.7% vs 97% *P* = 0.05	CTCAE ver. 3.0	GI 1.6% vs 1.1% GU 20% vs 10. 4% *P* = 0.02	IG-IMRT improved PSA control in high-risk group IGRT reduced urinary toxicity
**IGRT**								
Vargas [27] (William Beaumont)	2005 (*n* = 331)	PII 63–79.2 Gy CBCT, Portal	1.6 years	3D-CRT 70.2 Gy vs 72 Gy vs 73.8 Gy vs 75.6 Gy vs 77.7 Gy vs 79.2 Gy	NA	CTCAE ver. 2.0	GI 27% vs 21% vs 11% vs 8% vs 15% vs 18% #Group 2 vs Group 1, 17% vs 8% *P* = 0.035	Acute related to late toxicity Wider field elevated toxicity
**IG-IMRT**								
Vora [28] (Mayo)	2013 (*n* = 302)	Long-term follow-up US or fiducial	91 months	75.6 Gy (70.2–77.4)	9 years (77.4%/69.6%/53.3%)	CTCAE ver. 4.0	GI 2.3% GU 10%	Long-term results
Tomita [29] (Aichi CC)	2013 (*n* = 241)	Helical tomotherapy MVCT	35 months	74–78 Gy	NA	RTOG	GI 7.4%	
Eade [31] (Australia)	2013 (*n* = 101)	Dose escalation Fiducial and/or daily CBCT	21 months	78.3–84 Gy	NA	CTCAE ver. 3.0/IPSS	GI 2% GU 3%	>78 Gy IG-IMRT well tolerated

IG-IMRT = image guided IMRT, MSK = Memorial Sloan-Kettering Cancer Center, Aichi CC = Aichi Cancer Center Hospital, US = ultrasonography, CBCT = cone-beam computed tomography, NA = not available, CTCAE = Common Terminology Criteria for Adverse Events, RTOG = Radiation Therapy Oncology Group, IPSS = International Prostate Symptom Score, GI = gastrointestinal, GU = genitourinary, *5 years unless otherwise stated, L/I/H = low risk/intermediate risk/high risk groups, *n* = 11 vs 48 vs 28 vs 136 vs 75 vs 33, #Low risk group was treated for prostate only (Group 1) and other treated for seminal vesicle and prostate (Group 2).

Zelefsky *et al*. reported outcomes of 86.4 Gy for 186 image-guided IMRT (IG-IMRT) treatments with a median follow-up period of 2.8 years using the placement of fiducial markers and daily tracking by kilovoltage imaging of target positioning [26]. This technique is associated with an improvement in biochemical tumor control among high-risk patients and a lower rate of late urinary toxicity compared with a similar dose of IMRT. This group of patients was retrospectively compared with a similar cohort of 190 patients without fiducial markers (non-IGRT). The 3-year likelihood of Grade ≥2 urinary toxicity for IGRT and non-IGRT cohort was 10.4% and 20.0%, respectively (*P* = 0.02). Multivariate analysis identifying predictors of Grade ≥2 late urinary toxicity demonstrated that in addition to the baseline International Prostate Symptom Score (IPSS), IGRT was associated with significantly less late urinary toxicity compared with the non-IGRT group. The incidence of Grade ≥2 rectal toxicity was low in both treatment groups (1.0% and 1.6%, respectively; *P* = 0.81). No differences in PSA relapse-free survival outcomes were observed in low- and intermediate-risk patients when either treated with IGRT or not treated with IGRT. Nonetheless, in high-risk patients, a significant improvement (97% vs 77.5%, *P* = 0.05) was observed 3 years after treatment with IGRT compared with non-IGRT.

Vargas *et al*. reported a Phase II adaptive radiation therapy (ART) trial in 331 patients with a median follow-up period of 1.6 years [27]. Low-risk patients (PSA < 10, stage < T2a, Gleason score <7) received irradiation to the prostate alone (Group 1). All other patients, both intermediate and high risk, received irradiation to the prostate and seminal vesicles (Group 2). Grade 2 chronic rectal toxicity was experienced by 34 patients (10%; 9% experienced rectal bleeding, 6% proctitis, 3% diarrhea, and 1% rectal pain). Nine patients (3%) experienced Grade ≥3 chronic rectal toxicity (one Grade 4). The 2-year rates of Grade ≥2 and Grade ≥3 chronic rectal toxicity were 17% and 3%, respectively. No significant difference among dose levels was seen in the 2-year rate of Grade ≥2 chronic rectal toxicity. These rates were 27%, 15%, 14%, 17% and 24% for dose levels equal to or less than 72, 73.8, 75.6, 77.4 and 79.2 Gy, respectively (*P* = 0.3). Grade ≥2 chronic rectal bleeding was significantly greater in Group 2 than in Group 1, 17% vs 8% (*P* = 0.035).

Vora *et al*. reported [28] long-term disease control and chronic toxicity in 302 patients. Chronic toxicity was measured at the peak in symptoms and at the last visit. The median radiation dose delivered was 75.6 Gy (range, 70.2–77.4), and 35.4% of the patients received ADT. The patients were followed up until death or for 6–138 months (median, 91) for those alive at last evaluation. At last follow-up, only 0% and 0.7% of patients had persistent Grade ≥ 3 GI and GU toxicity, respectively.

Tomita *et al*. reported helical tomotherapy (HT) results for 241 patients with a median follow-up time of 35 months [29]. Late Grade 2–3 rectal toxicity was observed in 18 patients (7.4%). Age, the maximum dose for the rectum, V70 and V60 of the ≥ Grade 2 toxicity group were significantly higher than in the ≤ Grade 1 toxicity group (*P* = 0.000 93, 0.048, 0.0030 and 0.0021, respectively). None of the factors was significant in multivariate analysis. Nishimura *et al*. also examined late toxicity after HT in 117 patients [30] and found 7.7% cases of GI toxicity ≥ Grade 2 and 6.8% cases of GU toxicity ≥ Grade 2. They noted that these figures were higher than expected for IGRT–IMRT. These reports imply that the advanced IGRT techniques do not always lead to a reduction in late toxicity. Eade *et al*. used rectal dose constraints V65 < 17% and V40 < 35% [31]. The bladder dose goals were V65 < 25% and V40 < 50%. They concluded that doses >78 Gy delivered using daily image guidance and IMRT are well tolerated and that by 3 months, short-term side-effects are normalized in the majority of patients.

Thus far, IGRT stays only at the preliminary stage and does not lead to reduced toxicity. Concrete evidence may come from further research.

### Prognostic factors for the adverse reactions

#### Gastrointestinal toxicity

**(i) Rectal bleeding **Regardless of the type of radiation therapy, the most frequently considered functional endpoints in the published analyses are gastrointestinal (GI) toxicity complications and rectal bleeding (Table 4) [32–66]. Reported risk factors for late rectal bleeding after radiotherapy include hypertension [32], advanced age [32, 33], larger irradiated rectal volume [34, 35], a history of a prior abdominal surgical procedure [36–40], acute toxicity (including proctitis and mucous discharge) [17, 37–39, 43, 46–53), cardiac history [40], the use of ADT [41–45], hemorrhoids [54, 55], diabetes mellitus [56–59], inflammatory bowel disease (IBD) [60]. Acute toxicity is recognized as an independent significant factor confirmed in several trials. The question arises as to whether early interventions that lessen acute toxicity may also reduce the risk of late complications, or whether greater than expected acute toxicity may be an early indicator of a patient's hypersensitivity to radiotherapy.

**Table 4.** Reported risk factors for adverse reaction

Risk factors for late gastrointestinal (GI) symptom
** (1) Rectal bleeding**
Hypertension [32], Increased age [32, 33], Large rectum volume [34, 35]
Abdominal surgery [36–40], Acute symptom [17, 37–39, 43, 46–53], Cardiac history [40]
Androgen deprivation therapy (ADT) [41–45], Hemorrhoids [54, 55], Diabetes Mellitus [56–59]
Inflammatory bowel disease [60]
** DVH** (rectum)
V50 < 45–55%, V60 < 35–40%, V65 < 20–25%, V70 < 15–25%, V75 < 5–15% [15, 22, 36, 40, 45, 46, 50, 51, 53, 56, 59–65]
V40–60 Gy would be also important if prescribed 78 Gy or more [2, 36, 46, 51, 55, 59–68]
QUANTEC: V50 < 50%, V60 < 35%, V65 < 25%, V70 < 20%, V75 < 15% ⇒ Grade 2 < 15% [61]
*n = 0.09 (95% CI: 0.04–0.14); *m* = 0.13 (0.10–0.17); TD50 = 76.9 (73.7–80.1) Gy [61]
** (2) Incontinence**
Abdominal surgery [37, 38, 40, 69], Diabetes Mellitus [40], Cardiac history [40]
Antihypertensive drug (protective factor) [40, 69], Acute or prior (including mucous discharge, proctitis) [40, 72, 73]
Hemorrhoids [66], seminal vesicle irradiation [72], Previous bowel disease [69]
** DVH** (Anorectal–anal canal)
Anorectal V40 < 65–80% [37, 38], Mean dose < 45–50 Gy [6, 18, 36–38, 58, 59, 61–63, 66–71]
Anal canal <37 Gy [73–75], Anal sphincter lesion V35 <60% V40 < 40% [76]
** Risk factors for late genitourinary (GU) symptom**
ADT [37, 38], TURP [38], Hypertension [38], Pre-RT symptoms [38]
Acute symptom [17, 43], Increased age [82], Pre-RT GU medication [47]
** DVH** (Bladder)
Max dose <78 Gy to 80 Gy [17, 54, 80] V30 < 30 cm^3^, V82 < 7 cm^3^ [80]
QUANTEC: V65 ≤ 50%, V70 ≤ 35%, V75 ≤ 25%, V80 ≤ 15% RTOG 0415 recommendation [81]
** Risk factors for erectile dysfunction (ED)**
Pre-RT sexual function [23, 82], Increased age [47, 83, 87], Diabetes Mellitus [47, 87], ADT [47, 83, 87], Pre-RT PSA value [83]
** DVH** (Penile bulb)
V40 < 40% V50 < 20% [84], Median >52.5 Gy [85], V70 < 70% [88]
QUANTEC: Mean 95% < 50 Gy, D60–70 <70 Gy, D90 < 50 Gy ⇒ severe ED < 35% [88]

*Lyman–Kutcher–Burman normal tissue complication probability model, DVH = dose–volume histogram, QUANTEC = quantitative analysis of effects on normal tissue in the clinic.

Significant differences exist among studies in terms of techniques, procedures, definitions of the rectum (including filling, surface and wall), and the potential impact of set-up motion. Nevertheless, there are several well-established significant volume effects for partial irradiation to the rectum. The volume of the rectum receiving ≥60 Gy is consistently associated with a risk of Grade ≥2 rectal toxicity or rectal bleeding [36, 40, 45, 46, 50, 51, 56, 59–65). Several studies support a correlation between Grade 2–3 bleeding and both high (volume receiving >70 Gy [V70]) and intermediate (V50–V60) doses if a higher dose (>78 Gy) was prescribed [2, 36, 46, 51, 55, 59–65]. The conservative dose–volume constraints are V50 < 45–55%, V60 < 35–45%, V65 < 25%, V70 < 15–25% and V75 < 5–15%, although these constraints have yet to be validated as relatively safe [15, 22, 36, 40, 50–53, 59–65]. For typical dose–volume histograms (DVHs), the normal tissue complication probability (NTCP) models predict that following these constraints should limit Grade ≥2 late rectal toxicity to < 15% and the probability of Grade ≥3 late rectal toxicity to < 10% for prescriptions of up to 79.2 Gy in standard 1.8–2-Gy fractions. The parameters for the Lyman–Kutcher–Burman normal tissue complication probability model were estimated {*n* = 0.09 (95% CI: 0.04–0.14); *m* = 0.13 (0.10–0.17); and TD_50_ = 76.9 (73.7–80.1) Gy}. Clinicians should strive to minimize the V70 and V75 volumes below the recommended constraints without compromising tumor coverage. In other words, reducing V75 by only 5% (from 15% to 10%) has a significant impact on the complication probability, whereas reducing V50 from 50% to 45% makes relatively little difference for rectal bleeding [61]. Several authors proposed custom-made constraints based on generic and patient-specific risk factors. For example, an Italian group attempted to examine the influence of a prior abdominal surgical operation on the correlation of G2–G3 bleeding with a cholecystectomy [OR = 6.5, *P* = 0.002) and on a secondary correlation with an appendectomy (OR = 2.7, *P* = 0.10) [39, 59]. Next, [36, 51, 66] they proposed a modified constraint for bleeding V70 < 15% (V75 < 5%) for patients with a history of abdominal or pelvic surgical procedures, but V70 < 25% (V75 < 15–20%) otherwise.

**(ii) GI incontinence **According to Denham *et al*. [53], fecal urgency and bleeding have the highest impact on daily life (Table 4) [37–77]. Koper *et al*. [6] have shown that patients are more bothered by symptoms such as soiling, fecal loss, and mucus discharge rather than blood loss, urges, and bowel cramps. Reported risk factors for late incontinence are: a previous abdominal or pelvic surgical procedure [37, 38, 40, 69], diabetes mellitus [40], a history of cardiac problems [40], the use of antihypertensive drugs (a protective factor) [40, 69], prior or acute symptoms (mucous discharge, proctitis) [44, 72, hemorrhoids [66], seminal vesicle irradiation [72], and previous bowel disease [69].

Potential mechanisms involved in the development of incontinence could be the reduced absorption capacity of the rectal mucosa, which may be expected to have a large volume effect as well as neurovascular damage impairing the musculature surrounding the rectum. Several recent studies produced evidence of dose–volume relations for late rectal incontinence [36–38]. It was demonstrated recently that a DVH constraint of rectum V40 < 65% or V40 < 80% (or a mean rectal dose of < 45–50 Gy) reduces the risk of late incontinence [6, 18, 20, 36–38, 58, 59, 61–63, 66–71]. Although late incontinence is quite a rare side-effect in modern radiotherapy, the application of this constraint has the potential to reduce the risk to <2%. In addition, several authors found a link to acute adverse reactions of Grade 2 and 3, which correlates strongly with the mean dose; these data suggest that the reduction of the dose bath delivered to the whole rectum may have an impact on the risk of acute toxicity [37, 38, 74]. Detailed analysis of the subarea DVH could provide further insights into the incontinence risks [33, 38, 63, 73]. Heemsbergen *et al*. reported a subarea difference: for bleeding and a mucus loss, the strongest correlation was found for the dose delivered to the upper 70–80% of the anorectal region (*P* < 0.01) [73]. For soiling and fecal incontinence, they found the strongest association with the dose delivered to the lower 40–50% of the anorectal region. For example, the anal canal was contoured by taking the caudal 3 cm of the anorectal portion [38]; 53 Gy delivered to the anal surface was found to be an important constraint [75]. Al-Abany *et al*. also reported dose constraints: a dose V35 < 60% or V40 < 40% of the anal sphincter region volume for fecal leakage [76]. A recent study proposed more detailed dose constraints: 30 Gy delivered to the internal anal surface, 10 Gy to the external anal surface, 50 Gy to the puborectalis muscle, and 40 Gy to the levator ani muscles [68].

Nevertheless, the prevalence and severity of diarrhea and rectal bleeding after 3D-CRT have been reported to be reduced in the long run compared with 2D RT [5–16]. Yeoh *et al*. showed that urgency of defecation, the most frequent sequela of RT, is not improved by the 3D-CRT technique, and is more frequent compared with the 2D technique [77]. They compared the frequency of anomalies between 3D-CRT and 2D radiotherapy 2 years after treatment: increased stool frequency [55% vs 53%, *P* = not significant (n.s.)], urgency of defecation (72% vs 47%, *P* < 0.05), fecal incontinence (28% vs 26%, *P* = n.s.), and rectal bleeding (38% vs 42%, *P* = n.s.). In the IMRT era, we are awaiting the evidence of reduction of those figures by IMRT or more modern techniques.

### Genitourinary adverse reactions

Mild acute irritative urinary symptoms have been reported in several studies, whereas total urinary incontinence and other severe late urinary symptoms (i.e. urethral stricture) are rare.

ADT [37, 38], prior transurethral resection of the prostate (TURP) [38], hypertension [38], pretreatment GU complaints [38], the presence of acute GU toxicity [17, 43], age > 70 [82], and GU medications before IMRT [47] are risk factors of long-term urinary morbidity (Table 4) [37–38, 43, 47, 54, 70, 80–82].

In the case of the bladder, there is a clear dose effect when the whole organ is irradiated (i.e. for cystitis) [78]. On the other hand, in the case of prostate irradiation, the cranial portion of the bladder is generally spared, whereas the bladder neck and urethra are irradiated near the prescribed dose [80]. The lack of knowledge about the dose–volume modeling of bladder toxicity probably reflects the difficulties with accurate assessment of the amount of bladder wall that receives a certain dose. This is because large variations are observed in the bladder shape during treatment because of variable filling. Serial behavior was reported recently for late mild to severe toxicity [54], whereas serial–parallel behavior was reported for chronic moderate or severe urinary toxicity [80]. Both studies indicated that the fraction of bladder receiving >78–81 Gy is most predictive of late GU toxicity [17, 54, 80].

### Erectile dysfunction

ED is not an immediate side-effect of RT (Table 4) [23, 47, 80–90], and the occurrence of spontaneous erection before treatment (Table 4) [23, 47, 81–90] is the best predictor of preservation of erectile function sufficient for intercourse [81–83]. Other clinical predisposing factors are older age [47, 82], diabetes mellitus [47, 82], ADT [82, 83] and previous PSA level [83]. Most, but not all, studies find an association between ED and dosimetric parameters [83–88]. Wernicke *et al*. reported significant constraints of V50 < 20% and V40 < 40%, and median D30, D45, D60 and D75 [84]. Roach *et al*. reported that patients whose median penile bulb dose was >52.5 Gy had a greater risk of ED based on the RTOG 9406 trial data [85]. They updated those constraints in quantitative analysis of effects on normal tissue in the clinic (QUANTEC) to a mean dose of V95 < 50 Gy, D60–70 < 70 Gy and D90 < 50 Gy [88] and recommend the use of the International Index of Erectile Function (IIEF) [88, 90]. The target organ at risk is not likely to be the penile bulb but appears to be a surrogate for yet to be determined structure(s) necessary for erectile function [87, 88], such as the crura, vascular structures, or other penile components [89]. Coverage of the planned target volume should not be compromised, and the use of magnetic resonance imaging (MRI) is preferable to define the apex of the prostate, with consequent efficient sparing of the organs at risk [82–86, 89].

## DISCUSSION

There are many modalities in radiation therapy, which cause a range of incidences of late GI toxicity. Kim *et al*. analyzed 28 088 patients using the SEER data. The most common GI toxicity is GI bleeding or ulceration. GI toxicity rates are 9.3 per 1000 person-years after 3D-CRT, 8.9 per 1000 person-years after IMRT, 20.1 per 1000 person-years after proton therapy, and 2.1 per 1000 person-years for patients receiving conservative management. Radiation therapy is the most significant factor associated with an increased risk of GI toxicity (HR, 4.74; 95% CI, 3.97–5.66). Even after 5 years, the radiation group continues to experience significantly higher rates of new GI toxicity than the conservative management group (HR, 3.01; 95% CI, 2.06–4.39) [91].

The RTOG or CTCAE scoring system has been widely used for assessment of toxicity but not enough to meet the requirements, according to a recent radiotherapy outcome survey. This is because in these scoring systems, compliance-related symptoms (such as stool frequency) and proctitis-related symptoms (such as rectal bleeding) are combined into one overall score. This feature may result in a loss of information and may obscure the relation between dose–volume parameters and complications [43]. Accordingly, several trials added a patient self-assessment questionnaire to obtain detailed information on morbidity. In addition, longitudinal assessment may add more useful information than peak score analysis can [43, 63, 68]. Gulliforde *et al*. found that endpoint—stool frequency—statistically significant dose–volume constraints are only derived by a longitudinal definition of toxicity in the outcome analysis of the MRC RT01 trial [63]. By the same token, an apparent association exists between acute side-effects experienced during the course of radiotherapy and the development of late toxicity. Heemsbergen *et al*. noted such an association between acute and late GI toxicity and postulated that late effects are a direct consequence of the initial tissue injury, which is reflected in acute symptoms resulting from inflammation of normal tissue [77]. According to their report, the presence of diarrhea during treatment is associated with a higher risk of late Grade ≥2 toxicity in late proctitis. They found that acute toxicity during treatment often manifests as tenesmus and internal hemorrhoid inflammation, which are associated with a higher likelihood of late proctitis. In addition, acute urinary symptoms that manifest during radiotherapy are linked to an increased risk of late Grade 2 urinary adverse events. Kim *et al*. [92] reported the long-lasting nature of GU toxicity: Grade 2–4 GU toxicity attributable to radiation therapy persists 10 years after treatment and thereafter based on comparison of 60 134 patients who received radiation therapy with 25 904 who underwent observation.

High-dose irradiation and/or hormonal therapy result in excellent outcomes, not only in PSA control, but also in overall survival. Nguyen *et al*. reported good 5- and 10-year actuarial overall survival rates (no ADT plus 75.6 Gy, 87.3% and 72.0% respectively; and ADT plus 75.6 Gy, 92.3% and 72% respectively; *P* = 0.0035) [4]. We also obtained similar results: 70 Gy plus ADT achieve 91–93% of overall survival after 5 years [7, 93]. Therefore, we should pay attention to adverse effects and quality of life (QOL) rather than disease control because almost 90% of the patients after EBRT live longer than 5 (or 10) years.

Multiple health-related QOL studies have been conducted using the IPSS, IIEF, and the European Organization for Research and Treatment of Cancer Quality of Life Questionnaire for Prostate Cancer 25 items (QLQ-PR25) etc. Such comparison between radical prostatectomy, EBRT, BT, and combined approaches uncovers a link between observed toxicity and QOL. For example, Sanda *et al*. prospectively measured outcomes reported by 1201 patients and 625 spouses or partners at multiple centers before and after radical prostatectomy, BT or EBRT [94]. Adjuvant ADT is associated with worse outcomes across multiple QOL domains among patients receiving BT or radiotherapy. Patients in the BT group report long-lasting urinary irritation, bowel and sexual symptoms, and transient problems with vitality or hormonal function. Adverse effects of prostatectomy on sexual function are mitigated by nerve-sparing procedures. After prostatectomy, urinary incontinence is frequent, but urinary irritation and obstruction are improved, particularly in patients with a large prostate. No treatment-related deaths occurred in that study; serious adverse events were rare. Their results suggest that treatment-related symptoms are exacerbated by obesity, large prostate size, high PSA score and older age. Black patients report a lower degree of satisfaction with the overall treatment outcomes. Changes in QOL are significantly associated with the degree of outcome satisfaction among patients and their spouses or partners. However, there are several problems with the use of QOL questionnaires. For example, the IPSS is considered a major QOL questionnaire in the treatment of prostate cancer, but IPSS was constructed mainly for prostate hypertrophy symptoms. Thus, this questionnaire cannot evaluate adverse effects after prostatectomy (the IPSS of most patients improves after prostatectomy). Therefore, when it comes to comparison of different treatment methods, accurate QOL evaluation is a challenge.

The impact of age on prostate cancer outcomes was found not only in PSA control and survival but also in QOL in less aggressive prostate cancers in older men [95], independent of other clinical features. When adjusted for other covariates, age >70 years still correlates with decreased OS (HR, 1.56 [95% CI] 1.43–1.70 *P* < 0.0001) and with a decreased incidence of metastasis (HR, 0.72 [95% CI, 0.63–0.83], *P* < 0.0001) and prostate cancer-specific death (HR, 0.78 [95% CI, 0.66–0.92], *P* < 0.0001). Although the biological underpinnings of this finding remain unknown, stratification by age in future trials is warranted. Several reports show that adverse reactions occur more frequently in older patients [32, 33, 77]. In this context, major data provided by a clinical trial (i.e. a large randomized controlled trial) were based on the data from patients younger than 80 years of age.

There are several limitations to our study. First, we did not analyze BT (although there are plenty of data in the literature) because we focused on the changes in adverse effects as a result of the advancement of EBRT from 2D to IMRT and IGRT. Second, as a result of this we did not analyze particle therapy because of the limited use of this therapy (both proton and carbon ion) in patients with prostate cancer except for clinical studies. Finally, hypofractionated radiotherapy was also excluded from this analysis, even though there is a hypothesis that hypofractionation has a radiobiological advantage in prostate carcinoma because of the low α/β ratio. This topic—the influence of fractionation—is beyond the scope of this study and will be explored in future studies.

In conclusion, the focus of toxicity analysis following radiotherapy for prostate cancer patients is changing from rectal bleeding to total elaborate QOL assessment.

## References

[1] Viani GA, Stefano EJ, Alfonso SL (2009). Higher-than-conventional radiation doses in localized prostate cancer treatment: a meta-analysis of randomized, controlled trials. Int J Radiat Oncol Biol Phys.

[2] Diez P, Vogelius IS, Benzten SM (2010). A new method for synthesizing radiation dose-response data from multiple trials applied to prostate cancer. Int J Radiat Oncol Biol Phys.

[3] National Comprehensive Cancer Network Clinical Practice Guidelines in Oncology. http://www.nccn.org/professionals/physician_gls/pdf/prostate.pdf.

[4] Nguyen QN, Levy LB, Lee AK (2013). Long-term outcomes for men with high-risk prostate cancer treated definitively with external beam radiotherapy with or without androgen deprivation. Cancer.

[5] Dearnaley DP, Khoo VS, Norman A (1999). Comparison of radiation side-effects of conformal and conventional radiotherapy in prostate cancer: a randomized trial. Lancet.

[6] Koper PC, Jansen P, van Putten W (2004). Gastro-intestinal and genito-urinary morbidity after 3D conformal radiotherapy of prostate cancer: observations of a randomized trial. Radiother Oncol.

[7] Yoshioka Y, Suzuki O, Nishimura K (2013). Analysis of late toxicity associated with external beam radiation therapy for prostate cancer with uniform setting of classical 4-field 70 Gy in 35 fractions: a survey study by the Osaka Urological Tumor Radiotherapy Study Group. J Radiat Res.

[8] Kuban DA, Tucker SL, Dong L (2008). Long-term results of the M. D. Anderson randomized dose-escalation trial for prostate cancer. Int J Radiat Oncol Biol Phys.

[9] Zietman AL, DeSilvio ML, Slater JD (2005). Comparison of conventional-dose vs high-dose conformal radiation therapy in clinically localized adenocarcinoma of the prostate: a randomized controlled trial. JAMA.

[10] Peeters ST, Heemsbergen WD, Koper PC (2006). Dose-response in radiotherapy for localized prostate cancer: results of the Dutch multicenter randomized phase III trial comparing 68 Gy of radiotherapy with 78 Gy. J Clin Oncol.

[11] Al-Mamgani A, van Putten WL, Heemsbergen WD (2008). Update of Dutch multicenter dose-escalation trial of radiotherapy for localized prostate cancer. Int J Radiat Oncol Biol Phys.

[12] Dearnaley DP, Sydes MR, Graham JD (2007). Escalated-dose versus standard-dose conformal radiotherapy in prostate cancer: first results from the MRC RT01 randomised controlled trial. Lancet Oncol.

[13] Skwarchuk MW, Jackson A, Zelefsky MJ (2000). Late rectal toxicity after conformal radiotherapy of prostate cancer (I): multivariate analysis and dose-response. Int J Radiat Oncol Biol Phys.

[14] Pollack A, Zagars GK, Antolak JA (2002). Prostate biopsy status and PSA nadir level as early surrogates for treatment failure: analysis of a prostate cancer randomized radiation dose escalation trial. Int J Radiat Oncol Biol Phys.

[15] Michalski JM, Bae K, Roach M (2010). Long-term toxicity following 3D conformal radiation therapy for prostate cancer from the RTOG 9406 phase I/II dose escalation study. Int J Radiat Oncol Biol Phys.

[16] Beckendorf V, Guerif S, Le Prisé E (2011). 70 Gy versus 80 Gy in localized prostate cancer: 5-year results of GETUG 06 randomized trial. Int J Radiat Oncol Biol Phys.

[17] Zelefsky MJ, Levin EJ, Hunt M (2008). Incidence of late rectal and urinary toxicities after three-dimensional conformal radiotherapy and intensity-modulated radiotherapy for localized prostate cancer. Int J Radiat Oncol Biol Phys.

[18] Vora SA, Wong WW, Schild SE (2007). Analysis of biochemical control and prognostic factors in patients treated with either low-dose three-dimensional conformal radiation therapy or high-dose intensity-modulated radiotherapy for localized prostate cancer. Int J Radiat Oncol Biol Phys.

[19] Sharma NK, Li T, Chen DY (2011). Intensity-modulated radiotherapy reduces gastrointestinal toxicity in patients treated with androgen deprivation therapy for prostate cancer. Int J Radiat Oncol Biol Phys.

[20] Bekelman JE, Mitra N, Efstathiou J (2011). Outcomes after intensity-modulated versus conformal radiotherapy in older men with nonmetastatic prostate cancer. Int J Radiat Oncol Biol Phys.

[21] Sheets NC, Goldin GH, Meyer AM (2012). Intensity-modulated radiation therapy, proton therapy, or conformal radiation therapy and morbidity and disease control in localized prostate cancer. JAMA.

[22] Michalski JM, Yan Y, Watkins-Bruner D (2013). Preliminary toxicity analysis of 3-dimensional conformal radiation therapy versus intensity modulated radiation therapy on the high-dose arm of the Radiation Therapy Oncology Group 0126 prostate cancer trial. Int J Radiat Oncol Biol Phys.

[23] Alicikus ZA, Yamada Y, Zhang Z (2011). Ten-year outcomes of high-dose, intensity-modulated radiotherapy for localized prostate cancer. Cancer.

[24] Spratt DE, Pei X, Yamada J (2013). Long-term survival and toxicity in patients treated with high-dose intensity modulated radiation therapy for localized prostate cancer. Int J Radiat Oncol Biol Phys.

[25] Pederson AW, Fricano J, Correa D (2012). Late toxicity after intensity-modulated radiation therapy for localized prostate cancer: an exploration of dose-volume histogram parameters to limit genitourinary and gastrointestinal toxicity. Int J Radiat Oncol Biol Phys.

[26] Zelefsky MJ, Kollmeier M, Cox B (2012). Improved clinical outcomes with high-dose image guided radiotherapy compared with non-IGRT for the treatment of clinically localized prostate cancer. Int J Radiat Oncol Biol Phys.

[27] Vargas C, Wagner M, Indelicato D (2008). Image guidance based on prostate position for prostate cancer proton therapy. Int J Radiat Oncol Biol Phys.

[28] Vora SA, Wong WW, Schild SE (2013). Outcome and toxicity for patients treated with intensity modulated radiation therapy for localized prostate cancer. J Urol.

[29] Tomita N, Soga N, Ogura Y (2013). Preliminary analysis of risk factors for late rectal toxicity after helical tomotherapy for prostate cancer. J Radiat Res.

[30] Nishimura T, Yamazaki H, Okabe H (2012). Exceptionally high incidence of symptomatic Grade 2–3 late rectal toxicity in radiotherapy of prostate cancer in 2.2 Gy hypofractionation using image-guided intensity-modulated radiation therapy. Int J Radiat Oncol Biol Phys.

[31] Eade TN, Guo L, Forde E (2012). Image-guided dose-escalated intensity-modulated radiation therapy for prostate cancer: treating to doses beyond 78 Gy. BJU Int.

[32] Barnett GC, De Meerleer G, Gulliford SL (2011). The impact of clinical factors on the development of late radiation toxicity: results from the Medical Research Council RT01 trial (ISRCTN47772397). Clin Oncol (R Coll Radiol).

[33] Skwarchuk MW, Jackson A, Zelefsky MJ (2000). Late rectal toxicity after conformal radiotherapy of prostate cancer (I): multivariate analysis and dose-response. Int J Radiat Oncol Biol Phys.

[34] Jackson A, Skwarchuk MW, Zelefsky MJ (2001). Late rectal bleeding after conformal radiotherapy of prostate cancer. II. Volume effects and dose-volume histograms. Int J Radiat Oncol Biol Phys.

[35] Wachter S, Gerstner N, Goldner G (2001). Rectal sequelae after conformal radiotherapy of prostate cancer: dose-volume histograms as predictive factors. Radiother Oncol.

[36] Fiorino C, Fellin G, Rancati T (2008). Clinical and dosimetric predictors of late rectal syndrome after 3DCRT for localized prostate cancer: preliminary results of a multicenter prospective study. Int J Radiat Oncol Biol Phys.

[37] Peeters ST, Hoogeman MS, Heemsbergen WD (2006). Rectal bleeding, fecal incontinence, and high stool frequency after conformal radiotherapy for prostate cancer: normal tissue complication probability modeling. Int J Radiat Oncol Biol Phys.

[38] Peeters ST, Hoogeman MS, Heemsbergen WD (2005). Acute and late complications after radiotherapy for prostate cancer: results of a multicenter randomized trial comparing 68 Gy to 78 Gy. Int J Radiat Oncol Biol Phys.

[39] Valdagni R, Vavassori V, Rancati T (2012). Increasing the risk of late rectal bleeding after high-dose radiotherapy for prostate cancer: the case of previous abdominal surgery. Results from a prospective trial. Radiother Oncol.

[40] Defraene G, Van den Bergh L, Al-Mamgani A (2012). The benefits of including clinical factors in rectal normal tissue complication probability modeling after radiotherapy for prostate cancer. Int J Radiat Oncol Biol Phys.

[41] Sanguineti G, Agostinelli S, Foppiano F (2002). Adjuvant androgen deprivation impacts late rectal toxicity after conformal radiotherapy of prostate carcinoma. Br J Cancer.

[42] Feigenberg SJ, Hanlon AL, Horwitz EM (2005). Long-term androgen deprivation increases Grade 2 and higher late morbidity in prostate cancer patients treated with three-dimensional conformal radiation therapy. Int J Radiat Oncol Biol Phys.

[43] Schultheiss TE, Lee WR, Hunt MA (1997). Late GI and GU complications in the treatment of prostate cancer. Int J Radiat Oncol Biol Phys.

[44] Liu M, Pickles T, Agranovich A (2004). Impact of neoadjuvant androgen ablation and other factors on late toxicity after external beam prostate radiotherapy. Int J Radiat Oncol Biol Phys.

[45] Zelefsky MJ, Harrison A (1997). Neoadjuvant androgen ablation prior to radiotherapy for prostate cancer: reducing the potential morbidity of therapy. Urology.

[46] Vargas C, Martinez A, Kestin LL (2005). Dose-volume analysis of predictors for chronic rectal toxicity after treatment of prostate cancer with adaptive image-guided radiotherapy. Int J Radiat Oncol Biol Phys.

[47] Cahlon O, Zelefsky MJ, Shippy A (2008). Ultra-high dose (86.4 Gy) IMRT for localized prostate cancer: toxicity and biochemical outcomes. Int J Radiat Oncol Biol Phys.

[48] Capp A, Inostroza-Ponta M, Bill D (2009). Is there more than one proctitis syndrome? A revisitation using data from the TROG 96.01 trial.?. Radiother Oncol.

[49] Heemsbergen WD, Peeters ST, Koper PC (2006). Acute and late gastrointestinal toxicity after radiotherapy in prostate cancer patients: consequential late damage. Int J Radiat Oncol Biol Phys.

[50] Cozzarini C, Fiorino C, Ceresoli GL (2003). Significant correlation between rectal DVH and late bleeding in patients treated after radical prostatectomy with conformal or conventional radiotherapy (66.6-70.2 Gy). Int J Radiat Oncol Biol Phys.

[51] Fiorino C, Sanguineti G, Cozzarini C (2003). Rectal dose-volume constraints in high-dose radiotherapy of localized prostate cancer. Int J Radiat Oncol Biol Phys.

[52] O'Brien PC, Franklin CI, Poulsen MG (2002). Acute symptoms, not rectally administered sucralfate, predict for late radiation proctitis: longer term follow-up of a phase III trial—Trans-Tasman Radiation Oncology Group. Int J Radiat Oncol Biol Phys.

[53] Denham JW, O'Brien PC, Dunstan RH (1999). Is there more than one late radiation proctitis syndrome?. Radiother Oncol.

[54] Cheung MR, Tucker SL, Dong L (2007). Investigation of bladder dose and volume factors influencing late urinary toxicity after external radiotherapy for prostate cancer. Int J Radiat Oncol Biol Phys.

[55] Huang EH, Pollack A, Levy L (2002). Late rectal toxicity: dose-volume effects of conformal radiotherapy for prostate cancer. Int J Radiat Oncol Biol Phys.

[56] Herold DM, Hanlon AL, Hanks GE (1999). Diabetes mellitus: a predictor for late radiation morbidity. Int J Radiat Oncol Biol Phys.

[57] Akimoto T, Muramatsu H, Takahashi M (2004). Rectal bleeding after hypofractionated radiotherapy for prostate cancer: correlation between clinical and dosimetric parameters and the incidence of Grade 2 or worse rectal bleeding. Int J Radiat Oncol Biol Phys.

[58] Kalakota K, Liauw SL (2013). Toxicity after external beam radiotherapy for prostate cancer: an analysis of late morbidity in men with diabetes mellitus. Urology.

[59] Peeters ST, Lebesque JV, Heemsbergen WD (2006). Localized volume effects for late rectal and anal toxicity after radiotherapy for prostate cancer. Int J Radiat Oncol Biol Phys.

[60] Willett CG, Ooi CJ, Zietman AL (2000). Acute and late toxicity of patients with inflammatory bowel disease undergoing irradiation for abdominal and pelvic neoplasms. Int J Radiat Oncol Biol Phys.

[61] Michalski JM, Gay H, Jackson A (2010). Radiation dose-volume effects in radiation-induced rectal injury. Int J Radiat Oncol Biol Phys.

[62] Michalski JM, Bae K, Roach M (2010). Long-term toxicity following 3D conformal radiation therapy for prostate cancer from the RTOG 9406 phase I/II dose escalation study. Int J Radiat Oncol Biol Phys.

[63] Michalski JM, Winter K, Purdy JA (2005). Toxicity after three-dimensional radiotherapy for prostate cancer on RTOG 9406 dose Level V. Int J Radiat Oncol Biol Phys.

[64] Luo C, Yang CC, Narayan S (2006). Use of benchmark dose-volume histograms for selection of the optimal technique between three-dimensional conformal radiation therapy and intensity-modulated radiation therapy in prostate cancer. Int J Radiat Oncol Biol Phys.

[65] Gulliford SL, Foo K, Morgan RC (2010). Dose-volume constraints to reduce rectal side effects from prostate radiotherapy: evidence from MRC RT01 Trial ISRCTN 47772397. Int J Radiat Oncol Biol Phys.

[66] Fellin G, Fiorino C, Rancati T (2009). Clinical and dosimetric predictors of late rectal toxicity after conformal radiation for localized prostate cancer: results of a large multicenter observational study. Radiother Oncol.

[67] Smeenk RJ, Hopman WP, Hoffmann AL (2012). Differences in radiation dosimetry and anorectal function testing imply that anorectal symptoms may arise from different anatomic substrates. Int J Radiat Oncol Biol Phys.

[68] Smeenk RJ, Hoffmann AL, Hopman WP (2012). Dose-effect relationships for individual pelvic floor muscles and anorectal complaints after prostate radiotherapy. Int J Radiat Oncol Biol Phys.

[69] Fiorino C, Rancati T, Fellin G (2012). Late fecal incontinence after high-dose radiotherapy for prostate cancer: better prediction using longitudinal definitions. Int J Radiat Oncol Biol Phys.

[70] Boersma LJ, van den Brink M, Bruce AM (1998). Estimation of the incidence of late bladder and rectum complications after high-dose (70-78 Gy) conformal radiotherapy for prostate cancer, using dose-volume histograms. Int J Radiat Oncol Biol Phys.

[71] Mavroidis P, al-Abany M, Helgason AR (2005). Dose-response relations for anal sphincter regarding fecal leakage and blood or phlegm in stools after radiotherapy for prostate cancer. Radiobiological study of 65 consecutive patients. Strahlenther Onkol.

[72] Valdagni R, Rancati T, Fiorino C (2009). Predictive models of toxicity with external radiotherapy for prostate cancer: clinical issues. Cancer.

[73] Heemsbergen WD, Hoofeman MS, Hart GA (2005). Gastrointestinal toxicity and its relation to dose distributions in the anorectal region of prostate cancer patients treated with radiotherapy. Int J Radiat Oncol Biol Phys.

[74] Vavassori V, Fiorino C, Rancati T (2007). Predictors for rectal and intestinal acute toxicities during prostate cancer high-dose 3D-CRT: results of a prospective multicenter study. Int J Radiat Oncol Biol Phys.

[75] Buettner F, Gulliford SL, Webb S (2012). The dose-response of the anal sphincter region-an analysis of data from the MRC RT01 trial. Radiother Oncol.

[76] al-Abany M, Helgason AR, Cronqvist AK (2005). Toward a definition of a threshold for harmless doses to the anal-sphincter region and the rectum. Int J Radiat Oncol Biol Phys.

[77] Yeoh EK, Holloway RH, Fraser RJ (2009). Anorectal function after three- versus two-dimensional radiation therapy for carcinoma of the prostate. Int J Radiat Oncol Biol Phys.

[78] Burman C, Kutcher GJ, Emami B (1991). Fitting of normal tissue tolerance data to an analytic function. Int J Radiat Oncol Biol Phys.

[79] Fiorino C, Rancati T, Valdagni R (2009). Predictive models of toxicity in external radiotherapy: dosimetric issues. Cancer.

[80] Harsolia A, Vargas C, Yan D (2007). Predictors for chronic urinary toxicity after the treatment of prostate cancer with adaptive 3-dimensional conformal radiotherapy: dose–volume analysis of a phase II dose-escalation study. Int J Radiat Oncol Biol Phys.

[81] Viswanathan AN, Yorke ED, Marks LB (2010). Radiation dose-volume effects of the urinary bladder. Int J Radiat Oncol Biol Phys.

[82] Budäus L, Bolla M, Bossi A (2012). Functional outcomes and complications following radiation therapy for prostate cancer: a critical analysis of the literature. Eur Urol.

[83] Alemozaffar M, Regan MM, Cooperberg MR (2011). Prediction of erectile function following treatment for prostate cancer. JAMA.

[84] Wernicke AG, Valicenti R, DiEva K (2004). Radiation dose delivered to the proximal penis as a predictor of the risk of erectile dysfunction after 3-dimensional conformal radiotherapy for localized prostate cancer. Int J Radiat Oncol Biol Phys.

[85] Roach M, Winter K, Michalski JM (2004). Penile bulb dose and impotence after 3-dimensional conformal radiotherapy for prostate cancer on RTOG 9406: findings from a prospective, multi-institutional, phase I/II dose-escalation study. Int J Radiat Oncol Biol Phys.

[86] Fisch BM, Pickett B, Weinberg V (2001). Dose of radiation received by the bulb of the penis correlates with risk of impotence after 3-dimensional conformal radiotherapy for prostate cancer. Urology.

[87] Rivin Del Campo E, Thomas K (2013). Erectile dysfunction after radiotherapy for prostate cancer: a model assessing the conflicting literature on dose-volume effects. Int J Impot Res.

[88] Roach M, Nam J, Gagliardi G (2010). Radiation dose-volume effects and the penile bulb. Int J Radiat Oncol Biol Phys.

[89] Mangar SA, Sydes MR, Tucker HL (2006). Evaluating the relationship between erectile dysfunction and dose received by the penile bulb: using data from a randomised controlled trial of conformal radiotherapy in prostate cancer. Radiother Oncol.

[90] Rosen RC, Riley A, Wagner G (1997). The international index of erectile function (IIEF): a multidimensional scale for assessment of erectile dysfunction. Urology.

[91] Kim S, Shen S, Moore DF (2011). Late gastrointestinal toxicities following radiation therapy for prostate cancer. Eur Urol.

[92] Kim S, Moore DF, Shih W (2013). Severe genitourinary toxicity following radiation therapy for prostate cancer-how long does it last?. J Urol.

[93] Yamazaki H, Nishiyama K, Tanaka E (2008). Reduction of irradiation volume and toxicities with 3-D radiotherapy planning over conventional radiotherapy for prostate cancer treated with long-term hormonal therapy. Anticancer Res.

[94] Sanda MG, Dunn RL, Michalski J (2008). Quality of life and satisfaction with outcome among prostate-cancer survivors. N Engl J Med.

[95] Hamstra DA, Conlon AS, Daignault S (2013). PROSTQA Consortium Study Group. Multi-institutional prospective evaluation of bowel quality of life after prostate external beam radiation therapy identifies patient and treatment factors associated with patient-reported outcomes: the PROSTQA experience. Int J Radiat Oncol Biol Phys.

